# A Comprehensive Approach to Differentiating Systemic Lupus Erythematosus From Other Autoimmune Diseases Presenting With Optic Neuritis

**DOI:** 10.7759/cureus.83723

**Published:** 2025-05-08

**Authors:** Jason Peng, Nada Alrifai, Salvatore D DeSimone, Pietro M Gentile, Pamela Traisak, Marissa Karpoff, David Feinstein, Hala Eid, Joseph D DeSimone

**Affiliations:** 1 Medical School, Cooper Medical School of Rowan University, Camden, USA; 2 Rheumatology, Cooper University Hospital, Camden, USA; 3 Ophthalmology, Cooper University Hospital, Camden, USA; 4 Bone and Joint Institute, Cooper University Hospital, Camden, USA; 5 Orthopedic Surgery, Cooper University Health Care, Camden, USA; 6 Rheumatology, Cooper University Health Care, Camden, USA; 7 Rheumatology, Cooper University Hospital, Cooper Medical School, Camden, USA; 8 Ophthalmology, Wills Eye Hospital, Philadelphia, USA

**Keywords:** diagnosis, multiple sclerosis, neuromyelitis optica spectrum disorder, optic neuritis, systemic lupus erythematosus

## Abstract

Systemic lupus erythematosus (SLE) is a complex autoimmune disease with significant morbidity and mortality. Early diagnosis is crucial for effective management. Ocular manifestations, particularly optic neuritis (ON), have emerged as potential early signs of SLE. This systematic literature review analyzes 23 studies, including eight retrospective studies, five cross-sectional studies, two cohort studies, four case reports, one narrative review, and three systematic literature reviews, to investigate the use of ON in diagnosing SLE and differentiating it from other autoimmune diseases, most notably multiple sclerosis (MS) and neuromyelitis optica spectrum disorder (NMOSD). A comprehensive literature search was conducted by combining various keywords and MeSH terms, focusing on autoimmune diseases, ON, MS, NMOSD, and SLE. The final selection of 23 studies reflected a methodologically diverse body of literature, enhancing the review’s robustness. SLE-associated ON is rare but distinctive, occurring in 1% of SLE patients and often presenting bilaterally with severe visual impairment and intense pain. Recovery of visual acuity is less common in SLE-associated ON compared to idiopathic ON and multiple sclerosis. Diagnostic challenges arise due to overlapping clinical features with NMOSD and MS. Various studies have highlighted the significance of specific antibody profiles, such as anti-aquaporin-4 IgG, in distinguishing between these conditions. Additionally, neuroimaging findings, including MRI characteristics, play a pivotal role in differentiation. SLE patients may exhibit unique lesion patterns spanning multiple vertebral bodies. Dermatological manifestations, genetic factors, and blood biomarkers, such as semaphorins and complement levels, also offer diagnostic insights. This systematic review underscores the importance of integrating clinical, laboratory, and radiologic imaging data for precise diagnosis and differentiation of SLE, NMOSD, and MS when encountering ON in clinical practice. These findings contribute to the development of concrete diagnostic criteria for SLE using ON as an early symptom, facilitating faster diagnosis and more efficient management of this debilitating autoimmune disease.

## Introduction and background

Systemic lupus erythematosus (SLE) is a multisystem autoimmune disease with unknown etiology that causes widespread morbidity and mortality [1]. Because of its complex disease pathogenesis and heterogeneity of symptoms, it is difficult to diagnose and is not a reportable disease, making it expensive to accurately document for epidemiologic studies [1,2]. The global incidence of SLE is 5.15 (1.4-15.13) per 100,000 person-years. Mortality is caused by either organ failure, infection, or cardiovascular disease [2,3]. It takes an individual an average of two years to be diagnosed with SLE after the initial onset of symptoms [4,5]. The timeliness of an SLE diagnosis significantly impacts prognosis, as early detection can avert severe tissue damage [4]. This is especially true considering SLE progression often occurs before the onset of clinical symptoms [5]. This diagnostic delay is clinically significant, as early detection is critical to preventing irreversible organ damage and improving long-term outcomes [4,5]. This is especially true considering SLE progression often begins before overt clinical symptoms appear [5]. Previous studies have shown that ocular manifestations can emerge as a first sign of disease in up to one-third of SLE patients [6]. Among these manifestations, optic neuritis (ON), keratoconjunctivitis sicca, and episcleritis are commonly presenting symptoms of SLE progression [7-9]. ON is relatively rare in the context of SLE, occurring in just 1% of cases, reflecting isolated ON as the initial manifestation of SLE rather than as a part of broader central nervous system (CNS) involvement [6]. Its rarity and potential early appearance have led some researchers to explore the utility of ON as an early diagnostic indicator of SLE [6].

ON is an ocular neuropathy with potential devastating consequences, including decreased visual acuity, orbital and ocular pain, metamorphopsias, and even blindness [10-12]. ON is commonly indicative of underlying autoimmune demyelinating disorders such as multiple sclerosis (MS), neuromyelitis optica spectrum disorder (NMOSD), and myelin-oligodendrocyte glycoprotein antibody-associated disease (MOGAD) [13]. In fact, about 50% of patients with ON go on to develop neurological symptoms suggestive of MS, and those with more severe ON often develop NMOSD [14-16]. It is important to note that SLE can occur concurrently alongside other inflammatory disorders [14,15]. ON can also be caused by both infectious and non-infectious inflammation [17]. These underlying causes result in occlusive vasculitis of the small arterioles of the optic nerve and edema of the myelinated nerve sheath with ensuing axonal necrosis and inflammatory demyelination of the optic nerve [9].

Given the prevalence of other underlying causes of ON, this systematic review will illustrate a diagnostic approach to (1) distinguish ON resulting from an autoimmune etiology as opposed to viral or other origins and (2) refine the diagnosis to suggest SLE, another autoimmune disorder, or concurrent conditions. Specifically, serological, radiological, and clinical features across SLE, MS, and NMOSD will be evaluated to guide differential diagnosis. This comprehensive approach incorporates factors, including antibody profiles, MRI findings, cerebrospinal fluid (CSF) studies, disease duration, and neurological symptoms. This study is unique in its holistic approach to provide a contemporary and inclusive overview of the defining characteristics of SLE, MS, and NMOSD, particularly in cases where ON is implicated.

## Review

Methodology

Although this comprehensive literature review was not conducted in strict accordance with the Preferred Reporting Items for Systematic reviews and Meta-Analyses (PRISMA) guidelines, several key elements aligned with PRISMA standards and were incorporated to ensure methodological rigor. A structured and replicable search strategy, defined inclusion and exclusion criteria, and an independent assessment of study quality and risk of bias by multiple reviewers were utilized. The deviation from full PRISMA adherence was primarily due to the exploratory nature of the review and the specificity of its focus, which did not require formal registration or full adherence to reporting checklists typically associated with larger-scale systematic reviews. Still, details of the search process, study selection, and quality assessment are presented below to ensure transparency and reduce the potential for bias in the identification and analysis of relevant literature. Additionally, due to significant heterogeneity in study designs, populations, and outcome measures among the included studies, a quantitative synthesis such as a meta-analysis or meta-regression was deemed inappropriate. As such, a narrative synthesis was performed to qualitatively summarize and compare the diagnostic features across different etiologies of ON. This allowed for a more comprehensive descriptive analysis while acknowledging the methodological differences between the included studies.

A literature search for the identification of relevant studies was conducted on June 5, 2023, using the following electronic databases: PubMed (https://pubmed.ncbi.nlm.nih.gov/, accessed June 5, 2023) and Ovid Medline (accessed June 5, 2023).

The context of this review was to evaluate the current literature to compare the systemic and clinical diagnostic features between patients with SLE-associated ON and ON caused by other etiologies. The search strategy was to identify the most effective MeSH terms using the PubMed Search manager. This was done to determine if the MeSH terms produced articles that accurately fit the topic of the study. This was also done to find corresponding keyword equivalences to increase the sensitivity of the literature search. The MeSH terms included autoimmune disease, optic neuritis, multiple sclerosis, neuromyelitis optica, systemic lupus, ocular, idiopathic, diagnosis, complication, cause, features, and characteristics. Each of these was searched without date limitations. The search terms were then paired into the following: “autoimmune disease, optic neuritis, features,” “autoimmune disease optic neuritis features, multiple sclerosis,” “autoimmune disease optic neuritis features, neuromyelitis optica spectrum disorder,” “autoimmune disease optic neuritis features, systemic lupus erythematosus,” “underlying diagnosis and characteristics for optic neuritis,” “ocular conditions associated with systemic lupus erythematosus,” “systemic lupus erythematosus optic neuritis,” “ocular manifestation, systemic lupus erythematosus,” “association, optic neuritis,” “cause, optic neuritis,” “systemic lupus erythematosus, optic neuritis,” “optic neuritis, autoimmune disease,” “ocular complication, systemic lupus erythematosus,” “idiopathic disease, optic neuritis,” and “systemic lupus erythematosus, ocular.” None of these searches had any other filters applied to them, and all article types were included. The total number of articles identified was 2,954.

Approximately 10-20 articles from each search term were analyzed to determine if the article topics matched the topic of our review. After this process, “ocular manifestation, systemic lupus erythematosus,” “association, optic neuritis,” “cause, optic neuritis,” “systemic lupus erythematosus, optic neuritis,” “optic neuritis, autoimmune disease,” “ocular complication, systemic lupus erythematosus,” “idiopathic disease, optic neuritis,” and “systemic lupus erythematosus, ocular” were eliminated. Alongside these eliminations, duplicate articles were removed, leaving 2,621 articles. Further, “autoimmune disease optic neuritis features, multiple sclerosis” and “autoimmune disease optic neuritis features, neuromyelitis optica spectrum disorder” were eliminated due to their overlap with the search term “autoimmune disease, optic neuritis, features,” leaving 718 articles after putting the remaining topics into a PubMed search in a combined prompt with “OR” statements in between (PubMed automatically removed any duplicate publications at this stage).

**Figure 1:**
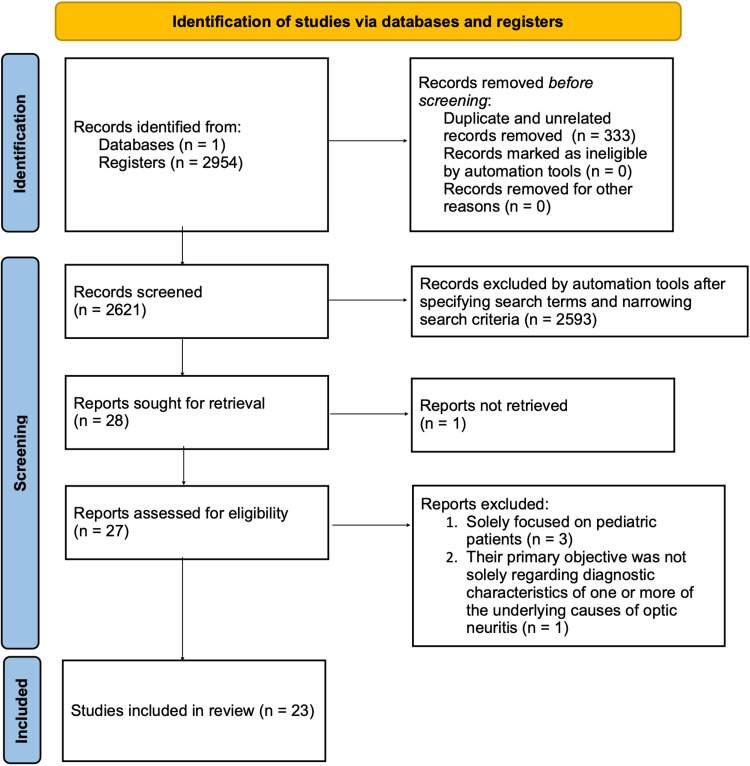
Flow diagram of the article selection process.

The search terms were then coded into PubMed as follows: “(autoimmune disease, optic neuritis, features) AND ((autoimmune disease optic neuritis features, systemic lupus erythematosus) OR (underlying diagnosis and characteristics for optic neuritis) OR (ocular conditions associated with systemic lupus erythematosus) OR (systemic lupus erythematosus optic neuritis)).” This generated 28 articles. After applying the following filters: (1) publication dates from January 1, 2012, to May 5, 2023; (2) humans only; (3) English only; and (4) Medline articles (preprints excluded), 25 articles were identified. Further, after reading through each of the articles, one was eliminated due to lack of access, and another was eliminated for deviating from the topic (i.e., if their primary objective was not solely regarding diagnostic characteristics of one or more of the underlying causes of ON), resulting in 23 articles for analysis. The remaining articles were screened by examining the reference lists of all articles they included. No articles were eliminated in this process. Inter-reviewer agreement during the full-text screening phase was assessed using a consensus-based review. Although a formal Cohen’s kappa statistic was not calculated, discrepancies between the authors were resolved through discussion until agreement was achieved. A PRISMA-style flow diagram showing the article selection process is illustrated in Figure 1.

The data extracted from the selected publications included any information about epidemiology, symptoms, treatment protocols, and diagnostic factors for SLE, MS, and NMOSD, along with the considerable limitations of the study. During the review process, the quality and risk of bias for each included study were independently assessed by three authors using a customized checklist based on the National Institutes of Health (NIH) Study Quality Assessment Tools [18]. The checklist evaluated criteria such as clarity of research objectives, specification of study population, validity and reliability of outcome measures, and control of key confounding variables. While a standardized scoring rubric was not applied, each study was independently reviewed by the authors, and final assessments of risk of bias (categorized as low, moderate, or high) were determined through group consensus before proceeding with data synthesis.

Our study did not require ethical board approval because it solely reviewed previously published studies and did not involve human participants or identifiable patient information.

Results

In total, 23 articles, including eight retrospective studies, five cross-sectional studies, two cohort studies, four case reports, one narrative review, and three systematic literature reviews regarding the presence of ON in patients with SLE, MS, NMOSD, or a combination of the three, were analyzed. All studies were conducted within either outpatient or inpatient hospital settings. The study type, country of study origin, year of publication, and population size are presented in Table 1. Additional information regarding the main findings and limitations of each study can be found in the Appendices.

**Table 1:** List of the 23 studies analyzed and their key features.

Author	Year	Country	Study type	Population
Zhang et al. [19]	2020	China	Retrospective study	225
Magro Checa et al. [20]	2013	The Netherlands	Review	N/A
Zhao et al. [21]	2016	China	Retrospective study	22
Nardone et al. [22]	2015	Austria	Case report	1
Kampylafka et al. [23]	2013	Greece	Cohort study	370
Fang et al. [24]	2020	Taiwan	Retrospective study	5,488
Siegel et al. [25]	2022	USA	Retrospective study	139
Martín-Nares et al. [26]	2019	Mexico	Retrospective study	12
Takahashi et al. [27]	2012	Japan	Cross-sectional study	47
Asgari et al. [28]	2013	Denmark	Review	1,493
Bibic et al. [29]	2019	Canada	Case report	2
Caroline Breis et al. [30]	2020	Brazil	Systematic review	N/A
Jain et al. [31]	2016	India	Retrospective study	64
Thabah et al. [32]	2019	India	Case report	8
Park et al. [33]	2014	Korea	Retrospective study	106
Xiao et al. [34]	2016	China	Retrospective study	64
Bruschi et al. [35]	2020	Italy	Narrative review	N/A
Sinnecker et al. [36]	2012	Germany	Cross-sectional study	28
Martin et al. [37]	2015	USA	Case report	434
Liang et al. [38]	2019	China	Cross-sectional study	1,715
Ostendorf et al. [39]	2019	Germany	Cross-sectional study	75
Moraitis et al. [40]	2019	London	Cross-sectional study	90
de Souza et al. [41]	2012	Brazil	Cohort study	36

The assessment of the risk of bias for each included study was conducted as described in the Methodology section. Overall, studies with a low risk of bias were described as having robust methodologies, adequate sample sizes, clear definitions, and appropriate statistical analyses. Studies with a high risk of bias had significant methodological concerns, including very small to small sample sizes, lack of a control group, or unclear measurement techniques. Ultimately, reviewers categorized nine studies as having a high risk of bias, 12 studies as having a moderate risk of bias, and two studies as having a low risk of bias. Detailed explanations for each of the assessments can be found in the Appendices. Especially for the studies with a high risk of bias, greater caution was applied when integrating conclusions from them, whereas findings from studies with moderate to low risk were prioritized in identifying diagnostic patterns. This approach strengthened the credibility of our conclusions, allowing them to be grounded in the most methodologically sound evidence available.

Discussion

Studies Included

This review synthesizes information from a variety of studies, emphasizing a heterogeneous selection of study designs. This diversity, while providing a broader perspective, introduces a main limitation of this study. Variations in methodological rigor, sample size, and data reporting may have limited the ability to draw uniform conclusions across studies. These differences may also contribute to inconsistencies in the interpretation of diagnostic features.

Epidemiology

SLE and SLE with transverse myelitis (SLE-TM) primarily affect women between 15 and 44 years old, with female-to-male ratios of 9:1 and 8:1, respectively [19-22]. Ethnic minorities, especially those of African and Asian descent, have a higher prevalence of SLE at 27% [21]. The average age of onset differs slightly between SLE cases with and without CNS involvement. Disease durations for SLE cases with CNS involvement are reported to be 5.7 ± 8.2 years, while those without CNS involvement have an average duration of 9.2 ± 7.8 years [23]. Of note, regional variation and socioeconomic disparities may significantly impact access to diagnostic tools and long-term outcomes of SLE-associated ON [2-4]. Specifically, settings with lower socioeconomic status may have delayed diagnosis and limited access to advanced imaging or specialist care, resulting in worse neurological outcomes and higher rates of permanent visual impairment or blindness [2-4].

MS is more common in individuals aged 35 years or older, with a ratio of 66:34 for the ≥35-year to 20-34-year age groups. The female-to-male ratio for MS is 73:27, which is more balanced compared to SLE [20,24]. MS has a higher prevalence among individuals of European descent [20]. From 2003 to 2015, there was a significant increase in MS prevalence, while the incidence remained relatively consistent [24,25].

NMOSD is also more common in individuals aged 35 years or older, with a ratio of 73:27 for the ≥35-year to 20-34-year age groups [24]. Approximately 41.7% of NMOSD cases followed a previous diagnosis of SLE or primary Sjögren’s syndrome (pSS) [26]. The sex ratio for NMOSD has shown a significant change over time. From 2001 to 2005, it was slightly more common in females aged 35 years or older. However, between 2011 and 2015, it became vastly more common in females in the same age group. Overall, NMOSD appears more frequently in females, with a ratio of 78:22 and an average age of 45.1 ± 14.6 years [24,26]. The disease course tends to be worse in patients younger than 12 years old [19]. The incidence and prevalence of NMOSD have increased over time, with higher rates observed among certain ethnic minorities, particularly individuals of Mexican or Japanese origin [24,26,27].

Optic Neuritis Features

SLE-associated ON is rare, accounting for fewer than 1% of cases [20]. It typically presents bilaterally, with involvement of the optic chiasm, severe visual impairment, and intense pain exacerbated by eye movements [20,22]. Visual acuity recovery to better than 20/25 is experienced by only 50% of SLE-associated ON patients, compared to 87% of idiopathic ON and MS patients [20]. Some cases of SLE-associated ON manifest unilaterally as thrombotic optic neuropathy associated with antiphospholipid antibodies [20].

In NMOSD-associated ON, the condition usually manifests as single or recurrent episodes, including bilateral simultaneous ON or sequential ON occurring rapidly one after another [28]. Among patients with recurrent ON, a study observed that 12% developed NMOSD and 14% developed MS within five years of the initial episode [28]. The risk of developing MS continued to increase beyond the five-year mark, while the risk for NMOSD plateaued [28]. The coexistence of SLE and NMOSD has been frequently reported, and MRI evidence of ON, TM, and elevated aquaporin-4 (AQP4) antibodies is used to confirm the presence of NMOSD in SLE patients [29].

In MS-associated ON, the condition typically presents unilaterally with reduced visual acuity, diminished color vision, mild disc swelling, mild pain upon eye movement, and an afferent pupillary defect [20]. Improvement of symptoms should begin within three weeks of onset [20]. Rarely, MS-associated ON can manifest bilaterally, leading to severe vision loss, photophobia, vitritis, neuroretinitis, and other complications [20]. About 28% of MS-associated ON cases are recurrent [25]. The features of ON between these three conditions are summarized in Table 2.

**Table 2:** Features of ON between SLE, NMOSD, and MS. ON = optic neuritis; SLE = systemic lupus erythematosus; NMOSD = neuromyelitis optica spectrum disorder; MS = multiple sclerosis; MRI = magnetic resonance imaging; TM = transverse myelitis; AQP4 = aquaporin-4

Feature	SLE-associated ON	NMOSD-associated ON	MS-associated ON
Laterality	Severe visual impairment, typically bilateral; some unilateral cases as thrombotic ON with antiphospholipid antibodies [20]	Bilateral simultaneous or sequential ON occurring rapidly one after another [28]	Typically unilateral [20]; rarely bilateral [20]
Visual recovery	~50% recover to better than 20/25 visual acuity [20]	Not specified	~87% recover to better than 20/25 visual acuity [20]
Pain	Intense; exacerbated by eye movement [20,22]	Not mentioned	Mild pain upon eye movement [20]
Associated features	May involve the optic chiasm; thrombotic ON associated with antiphospholipid antibodies [20]	Coexistence with SLE reported; diagnosis confirmed with MRI, ON, TM, and elevated AQP4 antibodies [29]	Mild disc swelling, diminished color vision, and afferent pupillary defect [20]; rarely: photophobia, vitritis, and neuroretinitis; symptoms should start to resolve within three weeks [20]

Neurological Symptoms

Neurological symptoms can indicate various conditions, including autoimmune diseases, infections, neoplasms, vascular conditions, trauma, and nutritional deficiencies [22]. One-third of SLE cases primarily present with neuropsychiatric involvement, while severe CNS involvement is seen in 4.3% of cases [23,30]. Symptoms range from epileptic seizures, strokes, myelopathy, psychosis, to aseptic meningitis. Mild CNS involvement may manifest as mild cognitive dysfunction, headaches, depression, anxiety, and paresthesias [23]. Epileptic seizures in SLE patients are often accompanied by glomerulonephritis, lupus nephritis, and posterior reversible encephalopathy syndrome on MRI [23]. Demyelination is almost always present in neuropsychiatric SLE and shares similar patterns with NMOSD [30].

Transverse myelitis (TM) is rare in SLE patients, occurring in 1-2% of cases [18,21]. It can manifest within the first five years after SLE diagnosis, often as a monophasic illness with poor recovery [19,22]. However, highly recurrent SLE-related longitudinally extensive transverse myelitis (LETM), a disease of contiguous, inflammatory spinal lesions that cause symptoms of paralysis, paresthesias, and urinary retention, is also reported [22]. NMOSD is characterized by ON and LETM, which are commonly present alongside acute brainstem syndrome and its symptoms of diplopia, intractable hiccup, nausea, and vomiting [22,26,30]. LETM in NMOSD typically manifests bilaterally with weakness and sensory disturbances, and there can be intervals between attacks as well [22,26]. Motor and sensory symptoms, loss of sphincter control, and tonic spasms can occur [26]. Gastrointestinal autoimmune diseases are commonly associated with MS but not with NMOSD [27].

In MS, immune cells infiltrate across the blood-brain barrier, promoting inflammation, demyelination, gliosis, and neuroaxonal degeneration of the CNS’s white matter, as apparent in the inflammatory demyelination often found in brain biopsies of MS patients [15]. This may result in asymmetrical, progressive myelitis, and short-segment spinal lesions in less than half of the spinal cord diameter, and may include nodular enhancements [15]. Further, cortical lesions are common, while thalamic/hypothalamic lesions are uncommon [15]. CSF analysis shows oligoclonal bands in >90% of patients, although CSF is usually normal [14]. In one study, myasthenia gravis has been reported to be slightly associated with MS at a rate of 0.9% (24/4,627 patients) [24].

Antibody Profiles

The primary biomarker indicating NMOSD is the presence of anti-aquaporin-4 (AQP4) IgG antibodies, but these may be absent in 5-40% of NMOSD patients [22,26,30-33]. As TM can be an initial symptom in both NMOSD and MS, examining serum AQP4-IgG levels in TM patients can aid in distinguishing between NMOSD and MS. NMOSD can coexist with other autoimmune disorders such as SLE, myasthenia gravis, and antiphospholipid syndrome [29,30,32]. In SLE patients who later develop NMOSD, 79% were found to have AQP4-IgG antibodies [30]. Another distinguishing marker is the presence of neuromyelitis optica immunoglobulin (NMO-IgG), which binds to AQP4 channels and indicates high-risk syndromes [22,28,34]. However, NMO-IgG antibodies are not consistently detected in NMOSD patients, although their presence predicts disease relapse [22,28,31,34]. In cases of LETM, the presence of AQP4-IgG antibodies is useful for distinguishing NMOSD (positive) from MS (negative) [22,32]. It is crucial to recognize that other antibodies, such as myelin-oligodendrocyte glycoprotein-IgG, may also be involved in NMOSD pathophysiology, especially in AQP4-IgG-negative patients [30]. Another factor in ruling out MS is the presence of acetylcholine receptor antibodies, found in 11% of NMOSD patients but not in MS or healthy individuals [29].

In SLE, the typical antibody profile includes antinuclear antibodies (ANAs), anti-single-stranded DNA (anti-ssDNA), anti-double-stranded DNA (anti-dsDNA), lupus anticoagulant (LAC), anticardiolipin antibodies (aCL), and anti-Sjögren’s syndrome-related antigen A/B (anti-SSA/SSB) antibodies [26,27,31,32]. It is important to note that these antibodies may be absent in some patients with clinically evident SLE, especially when the disease is in its early phases or presents atypically [26,32]. The absence of specific autoantibodies can complicate diagnosis and delay treatment, particularly in patients who present with neurologic symptoms suggesting the presence of overlapping autoimmune syndromes [26,32]. Generally, the presence of AQP4 antibodies is associated with seropositivity for SSA, SSB, ANA, and dsDNA antibodies [33]. The overlapping serological profiles of SLE, MS, and NMOSD underscore the diagnostic challenge of distinguishing a single one of these autoimmune disorders with the presence of autoantibodies alone. Additionally, MS patients may test positive for anti-Smith (Sm), ssDNA, and LAC antibodies, while NMOSD patients may be seropositive for aCL antibodies [27]. The interrelationships in autoantibody expression increase the risk of missclassification between demyelinating diseases and systemic autoimmune conditions. The presence of non-specific autoantibodies in MS or NMOSD, for instance, can mimic SLE-related neuroinflammation, requiring a careful and comprehensive diagnostic approach to avoid inappropriate management of these patients.

Imaging Findings

When suspecting a systemic disorder, a diagnostic approach typically involves cranial MRI scans, although full-body positron emission tomography scans are more suitable when there is suspicion of neoplasms [35]. The diagnostic criteria for differentiating between MS and NMOSD involve MRI. MRI also plays an integral role in distinguishing them from other neurological conditions [35]. The utilization of ultra-high-field strength (7-T) MRI, coupled with innovative 7-T T2*-weighted fast low-angle shot imaging, enables the identification of brain and spinal cord lesions, recognition of central vein signs, and detection of leptomeningeal enhancement. These advanced imaging techniques increase the sensitivity and specificity for diagnosing both MS and NMOSD [22,26,35,36].

In NMOSD, substantial and extensive plaques are associated with high AQP4 expression, primarily located in the periventricular and hypothalamic areas [26,31,34,36]. These plaques exhibit colocalized blood vessels in their periphery [31,36]. However, unspecified white matter lesions (WMLs) are also highly common in NMOSD [36]. Surprisingly, T2*-weighted images show a lack of hypointense rimming and, on rare occasions, the presence of crossing vasculature, despite the prominent vascular involvement in NMOSD [36].

In contrast to NMOSD lesions, MS plaques predominantly surround a small vein and exhibit hypointense rimming, suggesting neuroinflammation from vascular or unspecified WMLs [36]. T2*-weighted imaging reveals that 80% of MS lesions, with 19% originating from asymptomatic WMLs, are perivascular, indicating their predictive value for demyelinating diseases [36]. Furthermore, perivascular lesions, present in 92% of MS patients using 7-T MRI, are a requirement for classifying MS, as they demonstrate the presence of a vein within cerebral plaques [36]. Cortical lesions are also highly prevalent in MS [36]. The absence of MS-like lesions in asymptomatic patients confirms the unique morphology of MS plaques. Patients with SLE, on the other hand, have lesions spanning more than four vertebral bodies in approximately 60% of the cases [22].

Dermatological Symptoms

Pruritus, a neural pathway-induced sensation, has been identified as an early sign of NMOSD, often preceding weakness [32]. In one study, pruritus was observed in 28% of NMOSD patients, all of whom had NMO-IgG in their serum [34]. This sensation did not always align with the dermatomal distributions of the affected spinal cord segments, implying a spinal cord lesion. The precise mechanism underlying this sensation remains unclear. However, rodent studies suggest that trauma, tumors, and inflammation stimulate afferent neurons in the dorsal horn [34].

NMOSD also presents cutaneous and joint findings, with Raynaud’s phenomenon being the most common cutaneous manifestation [37]. Conversely, SLE commonly displays widespread dermatological manifestations, including malar rash, discoid rash, photosensitivity, and non-scarring alopecia, all of which are included in the current diagnostic criteria for SLE [32].

Genetic Involvement

The general transcription factor II-I (*GTF2I*) gene encodes transcription factor II-I (TFII-I), described primarily in mice as a crucial regulator of T and B-cell activation through vascular endothelial growth factor receptor 2 (*VEGFR2*) gene deregulation. In humans, its role has been characterized mainly in the regulation of other genes [38]. A robust genome-wide association study involving six East Asian cohorts confirmed a correlation between the *GTF2I* rs733666469 polymorphism and SLE occurrence. Another study suggested a potential association between the T allele of the *GTF2I* rs117026326 C/T polymorphism and SLE occurrence. However, further research is needed to establish the significance of this latter polymorphism [38]. Although the mechanism is not yet fully understood, the *GTF2I* rs117026326 polymorphism is also linked to NMOSD and MS [38]. Moreover, *GTF2I* polymorphisms, in general, have been found to be associated with rheumatoid arthritis, primary Sjögren’s syndrome, and other pathologies in Asian cohorts [38].

Bloodwork

Serum semaphorin 3A (SemaA3), recognized as an immunoregulatory molecule involved in oligodendrocyte regeneration and suppression of T cells, and its receptor neuropilin-1 have been implicated in the pathogenesis of SLE and other autoimmune diseases. Their levels are inversely correlated with SLE disease activity [30]. SLE patients are also prone to developing leukocytopenia and reduced complement levels [19,32]. Similarly, NMOSD patients may exhibit low serum complement levels [32].

Compared to healthy individuals, both MS and NMOSD patients have significantly elevated levels of low-density granulocytes (LDGs). However, the occurrence of LDGs in MS and NMOSD is not as frequent as in SLE patients [39]. In the study reported by Ossendorf et al., of the 23 healthy donors, none reached the 0.7% threshold (0.2%), while a majority of the 17 (0.9%) MS and 20 (2.1%) NMOSD patients did [39]. Nonetheless, the levels of LDGs in both MS and NMOSD do not match those found in SLE (4.3%) [39]. Although the exact mechanism remains unclear, the prevailing thought is that LDGs are a byproduct of ongoing inflammation rather than a part of the disease pathogenesis [39-41].

Other Findings

SLE-TM patients are more likely to experience fever compared to patients with SLE alone. The systemic inflammation caused by TM in SLE contributes to the higher incidence of fever [19]. A CSF white blood cell count exceeding 50 cells/µL indicates a possible infectious etiology, necessitating subsequent polymerase chain reaction analysis of CSF and additional viral serologies [22].

## Conclusions

SLE-associated ON, though rare, has diagnostic value as an early indicator of CNS involvement. Unlike ON associated with MS or NMOSD, SLE-associated ON tends to be bilateral, more severe, and less responsive to treatment. Through this systematic review, it is evident that no single clinical feature or diagnostic test is sufficient alone. Instead, a multidisciplinary approach, incorporating neuroimaging, serological markers such as AQP4-IgG, and patient demographics, provides the most robust framework for distinguishing among these autoimmune diseases. As a result, clinicians will be able to make more timely and precise diagnoses. Early recognition of ON in SLE not only facilitates prompt immunosuppressive treatment but may also alter the long-term neurological trajectory of patients. Additionally, emerging biomarkers, including LDGs and semaphorins, have shown early promise in identifying disease activity and may offer novel pathways for early diagnosis and monitoring in SLE-associated ON. Future research should aim to develop unified diagnostic criteria and explore novel biomarkers specific to SLE-associated ON. Further investigation of key biomarkers, as well as genetic and inflammatory mediators, could enhance early detection and lead to improved therapeutic strategies. Ultimately, integrating ocular symptoms such as ON into the diagnostic paradigm of SLE promises to enhance early detection, reduce morbidity, and improve quality of life for patients navigating this complex and often debilitating disease.

## Appendices

**Table 3:** Main findings and limitations of the 23 included studies. SLE = systemic lupus erythematosus; ON = optic neuritis; NMOSD = neuromyelitis optica spectrum disorder; MS = multiple sclerosis; TM = transverse myelitis; AQP4 = aquaporin-4; NPSLE = neuropsychiatric systemic lupus erythematosus; CNS = central nervous system; LETM = longitudinally extensive transverse myelitis; MOG = myelin-oligodendrocyte glycoprotein; pSS = primary Sjögren’s syndrome

Author:	Year	Country	Study type	Population	Main findings	Limitations
Zhang et al. [19]	2020	China	Retrospective study	225	SLE-TM patients have a higher frequency of fever and significantly higher rates of positive anticardiolipin and lupus anticoagulant in comparison to SLE patients without neuropsychiatric symptoms. 48.89% (22/45) of SLE-TM patients met the criteria for NMOSD. NMOSD patients had a lower incidence of rash, serositis, and renal disorder. 61.11% (11/18) of NMOSD patients were AQP4-IgG seropositive. Factors associated with poor outcome in SLE-TM patients included younger age, fever, initial severe myelitis, long spinal segment involvement, and higher C-reactive protein levels	Retrospective and single-center setting, which may impact the generalizability of the findings
Magro Checa et al. [20]	2013	The Netherlands	Review	N/A	Demyelination in NPSLE is rare, although prevalence depends on the criteria employed. There are no tests to distinguish MS and NPSLE. Instead, a combination of clinical, laboratory, and imaging tests may be used to distinguish the two diseases. Usually, long-term follow-up is required to make a diagnosis. TM and ON are the most common demyelinating syndromes in SLE. These must be differentiated from MS and NMO when seen clinically. -aPL antibodies are associated with SLE-TM. The pathogenesis is unclear. Immunosuppressive treatment should be started immediately in inflammatory demyelinating disorders of the CNS other than MS and those related to SLE (i.e., TM, ON, and NMO)	Replications of the study with a larger sample size in both patients with MS and NPSLE to determine the clinical significance. More information and research are needed regarding advanced MRI techniques in NPSLE and MS, distinguishing the two from each other
Zhao et al. [21]	2016	China	Retrospective study	22	HLA-B27 prevalence in ON patients was 5.4%, similar to the general Chinese population. HLA-B27-positive ON patients presented with recurrent episodes and severe visual impairment at onset. 72.7% of HLA-B27-positive ON patients had involvement of bilateral eyes. 13.6% of HLA-B27-positive ON patients were diagnosed with ankylosing spondylitis (AS). Bilateral sacroiliitis was confirmed in over 50% of HLA-B27-positive ON patients screened with pelvic CT. Some HLA-B27-positive ON patients had positive results for accompanying autoantibodies	The study had a retrospective design and a small sample size. Prevalence of HLA-B27 in the normal Chinese population was not analyzed. Diagnosis of certain autoimmune disorders relies on specific criteria without detailed information on their application for each patient. Lack of long-term follow-up data
Nardone et al. [22]	2015	Austria	Case report	1	Presents a rare case of LETM involving the cervical and thoracic spinal cord in an SLE patient. This is significant because only 37 cases of LETM in SLE patients have been previously described. An updated review of the diagnostic, management, and prognostic criteria of LETM in SLE was provided	Information on this condition in general is limited. It is a case study of one patient, so the generalizability of the findings is limited
Kampylafka et al. [23]	2013	Greece	Cohort study	370	The prevalence of major CNS events in SLE patients was 4.3%, with an annual incidence of 7.8 events per 100 person-years in newly diagnosed SLE patients. Major CNS manifestations of SLE include chorea, aseptic meningitis, psychosis, seizures, myelopathy, demyelinating syndrome, acute confusional state, and strokes. 4.32% (16/370) of SLE patients had CNS events, including seizures, strokes, myelopathy, optic neuritis, aseptic meningitis, and acute psychosis. No patient had overt cognitive deficits, and the overall mortality rate in the three-year follow-up period was 0.5%. CNS involvement was observed in 44% of SLE patients at the time of diagnosis. 25% had a relapse of the initial CNS manifestations within a three-year period. 13% of patients had concurrent CNS manifestations. SLE with CNS involvement is associated with arthritis, glomerulonephritis, hematologic disorders, and the use of corticosteroids. They also have higher hospitalization rates in comparison to SLE patients without CNS involvement	A single-center study. Relatively short follow-up period (three years)
Fang et al. [24]	2020	Taiwan	Retrospective study	5,488	The incidence of MS remained relatively stable over the years, while the incidence of NMO showed a steep increase during the study period of 2001 to 2015. Both MS and NMO showed a female preponderance in the study population. The most common autoimmune comorbidities for MS were Sjögren’s syndrome and systemic sclerosis, while for NMO, they were Sjögren’s syndrome and SLE. Taiwan transitioned from a low- to medium-risk zone for MS prevalence during the study period, although the incidence remained stable. The apparent increase in NMO incidence is attributed to increased awareness of this condition. The distinct autoimmune comorbidity profiles observed for MS and NMO suggest different underlying immunopathogenetic mechanisms for these conditions	The diagnosis of individual cases could not be determined, while the awareness of NMO as a distinct clinical entity was relatively recent. Thus, it is possible that some cases of NMO were misdiagnosed and misclassified as MS, especially during the early periods of the study. The date of disease onset was difficult to decipher when administrative data were repurposed for research. As such, the date of diagnosis was used instead. There is some lag between the two dates. The list of comorbidities is not exhaustive, and investigations are limited in that only the comorbidities already present before the diagnosis of MS or NMO were included. The results might be biased if there exists a fixed temporal sequence between MS or NMO and the comorbidities
Siegel et al. [25]	2022	USA	Retrospective study	139	139 patients with first episodes of ON associated with MS, AQP4-ON, and MOG-ON were analyzed. MOG-ON patients had the highest recurrence rate (88%) in comparison to MS-ON (28%) and AQP4-ON (56%) patients. Patients with AQP4-ON had the highest mean visual functional system scores (4.3) in comparison to MS-ON (2.0) and MOG-ON (2.8) patients. Atypical ON (AQP4-ON and MOG-ON) is more severe and associated with autoimmune antibody disorders like NMOSD. Accurate diagnosis of ON requires understanding each type’s variable characteristics	The characteristics and long-term prognostic criteria of MOG-ON are controversial and must be studied further
Martín-Nares et al. [26]	2019	Mexico	Retrospective study	12	The median time from the diagnosis of SLE/pSS to the first neurological event of NMOSD was 48 months. Patients experienced various neurological attacks associated with NMOSD, including acute TM, ON, cerebral syndrome, area postrema syndrome, brainstem syndrome, and cerebellar syndrome. All patients with SLE had positive ANA. Most patients with pSS had positive anti-RO/SSA antibodies. All patients with NMOSD had AQP4-IgG. Most patients with NMOSD achieved complete or partial remission using IV methylprednisolone, plasma exchange therapy, and immunosuppressants. Some experienced sequelae and disabilities such as motor impairment, sensory disturbances, neurogenic bladder, and visual impairment. When comparing NMOSD patients with SLE and NMOSD patients with pSS, those with SLE were more frequently treated with cyclophosphamide for remission and had a higher frequency of coexisting autoimmune diseases. Those with pSS had a trend for active disease at NMOSD onset. There was no significant difference in age at NMOSD onset, gender, clinical features, radiological features, or outcomes. The coexistence of NMOSD with SLE or pSS is a true overlap syndrome and not a manifestation of SLE or pSS itself	Some clinical and laboratory data could be missing due to the retrospective nature of the study. The small sample size prevents a thorough analysis of the possible differences between SLE/NMOSD and pSS/NMOSD. The patient demographic was mostly Hispanic
Takahashi et al. [27]	2012	Japan	Cross-sectional study	47	SLE-associated antibodies (i.e., anti-Sm, anti-ssDNA, and lupus anticoagulants) were only identified in MS patients. NMO showed a higher frequency of other autoreactive antibodies, such as RF, SS-A, aTG, and aTPO. 24.3% of MS patients had concurrence with other autoimmune diseases, the most common being Hashimoto thyroiditis. 60% of NMO patients had concurrent autoimmune diseases, Sjogren’s syndrome and Graves’ disease being the most common. Age of onset, disease duration, and expanded disability status scale (EDSS) did not show significant differences between autoreactive antibody-positive (AR(+)) and (AR(-)) MS patients. AR(+) MS cases had significantly fewer periventricular lesions in comparison to AR(-) MS cases. All of these suggest different underlying pathogenetic mechanisms for AR(+) MS and AR(-) MS	There is a lack of significant differences in clinical features, such as age of onset, disease duration, and EDSS, between AR(+) and AR(-) MS patients. Small sample size. Replication in larger cohorts is required to validate these findings and determine their functional significance
Asgari et al. [28]	2013	Denmark	Review	1,493	The discovery of a relapsing form of NMO led to the separation of NMO into two subtypes. AQP4-IgG is a key feature of NMO. NMO is recognized as an inflammatory demyelinating disease of the CNS that is distinct from MS and other inflammatory demyelinating diseases. NMO-IgG and AQP4-IgG from NMO selectively bind to AQP4 in astrocytic foot processes at specific locations in the CNS, inducing their effects. NMO and MS are distinct entities that have differing clinical, neuroimaging, immunological, and histopathological characteristics	Data sources were the DNPR and the clinical departments. As such, the number of MS cases was fewer than expected, raising issues of incomplete ascertainment. Of the selected high-risk MS group who fulfilled the inclusion criteria, 35 received natalizumab, which may hinder the diagnosis of NMO. The retrospective design of the clinical study may limit generalizability. The sensitivity and specificity of the assay to detect AQP4 antibodies were limited
Bibic et al. [29]	2019	Canada	Case report	2	Concurrent autoimmune diseases must always be considered with NMOSD. Plasmapheresis, steroids, and rituximab received by an NMOSD patient with concurrent hypothyroidism, SLE, and MG, and had moderate improvement but had SLE-related complications. IVIG, plasmapheresis, rituximab, and cyclophosphamide were received by a patient with ocular MG who developed NMOSD symptoms and had limited improvement with febrile neutropenia. AQP4-IgG-associated NMOSD may have shared autoimmune pathophysiology and genetic susceptibility with NMOSD, MG, and SLE. The coexistence of SLE and NMOSD is frequently reported, indicating a potential relationship between the two. Early diagnosis and treatment have better outcomes for NMOSD and concurrent autoimmune diseases	It is a case study of two patients, so the generalizability of the findings is limited
Caroline Breis et al. [30]	2020	Brazil	Systematic review	N/A	Some studies report the development of MOGAD features in certain SLE patients, but this is not universally observed. There are variations in results. NMOSD is associated with SLE and other autoimmune diseases. This association is strong in AQP4-IgG-seropositive NMOSD in comparison to MOG-IgG-seropositive NMOSD	The available data on the association between MOGAD and SLE is limited. Findings are inconsistent throughout studies, so more research on the connection between MOGAD and SLE is needed
Jain et al. [31]	2016	India	Retrospective study	64	LETM is very heterogeneous, with diverse clinical features, etiologies, and outcomes. It is important to distinguish between LETM and NMO, since not all LETM patients with ON have NMO. The diagnosis of NMO should be made after ruling out other causes of LETM. The presence of NMO antibodies predicts an increased risk of recurrence or conversion to NMO	The retrospective design of the clinical study may limit generalizability. The study was conducted at a single tertiary care center. The cause of LETM was unable to be determined in some patients despite thorough investigation
Thabah et al. [32]	2019	India	Case report	8	NMOSD is characterized by LETM, optic neuritis, and AQP4-IgG seropositivity. It may involve more brain regions. SLE may also involve neurological symptoms. SLE-TM usually involves fewer spinal cord segments, while LETM is rare but seen in NMOSD. Patient responds well to IV pulse methylprednisolone and plasma exchange with maintenance of low-dose prednisolone, azathioprine, and hydroxychloroquine. This is yet another case of NMOSD as an initial manifestation of SLE. In these cases, patients have LETM, are AQP4-IgG seropositive, and have serological evidence of SLE, including having ANA and anti-Sm antibodies	It is a case study of one patient, so the generalizability of the findings is limited
Park et al. [33]	2014	Korea	Retrospective study	106	18.9% (20/106) of NMOSD patients were positive for anti-Ro/SSA antibodies. Among the NMOSD patients who were positive for AQP4-IgG, 37.5% had SSA-IgG. The presence of SSA-IgG was highly associated with AQP4-IgG in NMOSD patients (90.0%). SSA-IgG was associated with systemic autoimmune diseases, like Sjögren’s syndrome and SLE, as well as the presence of ANA and anti-dsDNA-IgG. There are no significant differences in clinical features, disease duration, attack number, and annualized relapse rate between the SSA-IgG-seropositive and seronegative individuals of the AQP4-IgG-seropositive group	Retrospective design. Replication in larger cohorts is required to validate these findings and determine the clinical significance of SSA-IgG in NMOSD patients and the relationship between autoimmunity and NMO pathogenesis
Xiao et al. [34]	2016	China	Retrospective study	64	28.13% (18/64) of NMO patients had persistent, intractable pruritus with paresthesia in the affected areas without rash or obvious systemic causes. This episode occurred at the time of relapse and was not present during remission. This suggests that pruritus is not rare in NMO and may be a key feature of the disease. Spinal MRI showed lesions in all NMO patients with pruritus. 13 patients also had brain lesions. The anatomical distribution of pruritus did not always align with the dermatomal distributions of the affected spinal cord segments, suggesting other underlying mechanisms. Pruritus was partially relieved with IV methylprednisolone and oral corticosteroids	Retrospective study at one tertiary care facility. Potential bias as data was collected from patient telephone reviews
Bruschi et al. [35]	2020	Italy	Narrative review	N/A	7-T MRI allows for increased sensitivity and specificity for lesion detection and characterization of the brain and spinal cord, aiding in the diagnosis and understanding of MS. Identification of the central vein sign and leptomeningeal enhancement, both key imaging features of MS, are enhanced by 7-T MRI, allowing for easier differentiation. 7-T MRI can provide insights through metabolic imaging, improving our understanding of MS pathophysiology	Limited information on this topic, in general. There are currently no studies regarding acute demyelinating encephalopathy on 7-T MRI
Sinnecker et al. [36]	2012	Germany	Cross-sectional study	28	White matter lesions in NMOSD appeared non-specifically and infrequently around blood vessels, while MS plaques were the opposite. MS plaques commonly had hypointense rims, which were rarely detectable in NMOSD. Cortical pathology was absent in NMOSD, while 39% of MS patients had cortical lesions. The distribution pattern of white matter lesions was different between NMOSD and MS patients. NMOSD lesions were mainly located in the deep white matter. MRI features of white matter and the absence of cortical gray matter findings can be used to differentiate NMOSD from MS. The higher signal/noise ratio and greater anatomical details of 7-T MRI allow for better visualization of perivascular localization of MS plaques and detection of cortical pathology	Further research is needed to validate these findings and determine the clinical utility of such findings. Small sample size. The study needs to be replicated in larger cohorts
Martin et al. [37]	2015	USA	Case report	434	NMO is characterized by ON, longitudinal myelitis, and the presence of AQP4-IgG. The patient in this case report had longitudinal myelitis and AQP4-IgG, indicating a high risk of relapse or progression to full-blown NMO. Cutaneous findings in NMO are uncommon but may occur. This paper reports 10 cases, including this case report, of cutaneous findings in NMO. The most common cutaneous manifestation was Raynaud’s phenomenon. Clinical examination of this patient showed violaceous plaques and papules on the forehead and cheeks, as well as erythema and fissuring on the hands, synovitis in multiple joints, and longitudinal myelitis on imaging studies. Immunosuppressive therapy improved the cutaneous lesions. Serological testing showed inflammation and NMO-IgG seropositivity. AQP4-IgG may be produced in response to solid tumors, considering the widespread distribution of AQP4-IgG	It is a case study of one patient, so the generalizability of the findings is limited
Liang et al. [38]	2019	China	Cross-sectional study	1,715	There is a significant genetic association between the variant rs117026326 and NMOSD, but not with MS. The risk T allele of rs117026326 was found to increase the risk of NMOSD. There was no significant association between GTF2I MRNA expression and genotypes at the variant. NMOSD is distinguishable from MS by the seropositivity of AQP4-IgG and a variety of HLA and non-HLA polymorphisms. It is also more common in non-white populations, particularly in East Asian populations	This study has a small number of cases
Ostendorf et al. [39]	2019	Germany	Cross-sectional study	75	Low-density granulocytes (LDGs) were significantly more common in MS and NMOSD patients compared to healthy and SLE patients. A majority of NMOSD and MS patients had LDGs above the 0.7% threshold, while none of the healthy donors were above the threshold. NMOSD patients who did not receive continuous immunosuppressive treatment had significantly more LDGs compared to those who did. This was the first study to show the presence of LDGs in MS and NMOSD, suggesting the role of LDGs in these neuroinflammatory conditions. Before, LDGs were only linked with SLE. LDG nuclear morphology ranged from segmented to rounded	The exact role of LDGs in the pathogenesis of MS and NMOSD is unclear. Small cohort size. More longitudinal cohorts are required to make a definitive conclusion
Moraitis et al. [40]	2019	London	Cross-sectional study	90	5.56% (5/90) of jSLE patients tested positive for AQP4-IgG. All five had neurological involvement, mainly TM and ON. AQP4-IgG-positive patients were more likely to have neurological symptoms and less likely to have dermatological manifestations and detectable anti-dsDNA antibodies. All jSLE patients should be systematically screened for AQP4-IgG to determine the risk of neurological involvement	Retrospective study. Potential bias by case severity, as patients were included from two tertiary centers
de Souza et al. [41]	2012	Brazil	Cohort study	36	2.8% (1/36) of women with primary antiphospholipid syndrome tested positive for anti-nucleosome (anti-NCS/chromatin) antibodies. This one patient developed features of SLE, including polyarthritis, lymphopenia, ON, and MS-like lesions. They also developed an autoantibody profile suggestive of SLE. The low frequency of anti-NCS/chromatin antibodies in primary APS suggests an association between their presence and the development of SLE features	Small sample size. Larger, multicenter, and longitudinal studies are needed to confirm these findings and to discover the mechanisms linking anti-NCS/chromatin antibodies, APS, and SLE

**Table 4:** Assessment of the risk of bias for all 23 included studies.

Study	Year	Country	Study type	Population	Risk of bias	Reasoning
Zhang et al. [19]	2020	China	Retrospective study	225	Moderate	The study uses a retrospective design, which often faces issues with recall bias and the inability to control for all confounding variables. While the sample size is large (225 patients), the retrospective design means that causality cannot be inferred, and there is a risk of incomplete data collection or measurement bias. The study design and methodology likely introduce moderate bias
Magro Checa et al. [20]	2013	The Netherlands	Review	N/A	High	This is a review study, which inherently has a higher potential for bias due to selective inclusion of studies, lack of standardized criteria for study selection, and potential cherry-picking of data. Reviews can also suffer from a lack of transparency regarding the methodology, leading to potential bias
Zhao et al. [21]	2016	China	Retrospective study	22	High	With a very small sample size (22 patients), this study has a high risk of bias due to limited generalizability and potential for statistical instability. Additionally, retrospective designs often suffer from missing data, recall bias, and the inability to control for confounders, making the findings less reliable
Nardone et al. [22]	2015	Austria	Case report	1	High	As a case report with only one patient, this study lacks generalizability and does not allow for meaningful statistical analysis. The findings are very specific to the individual patient and may not apply to a larger population, leading to a high risk of bias
Kampylafka et al. [23]	2013	Greece	Cohort study	370	Low	This cohort study has a relatively large sample size (370 patients), and cohort studies generally provide more robust evidence than retrospective studies or case reports. The sample size is large enough to provide a reasonable estimate of the association, and the study is less prone to selection bias compared to smaller studies
Fang et al. [24]	2020	Taiwan	Retrospective study	5,488	Moderate	Although the sample size is large (5,488), retrospective studies still face limitations such as recall bias and confounding factors. However, the large population size helps mitigate some of the potential biases, and the study likely provides more reliable findings than studies with smaller samples
Siegel et al. [25]	2022	USA	Retrospective study	139	Moderate	A sample size of 139 is moderate, and while retrospective studies have limitations (e.g., potential confounding and measurement bias), the number of patients involved makes it relatively stronger. Still, causality cannot be determined, and retrospective studies often suffer from incomplete or biased data collection
Martín-Nares et al. [26]	2019	Mexico	Retrospective study	12	High	A very small sample size (12 patients) increases the risk of bias, as it may not be representative of the broader population. Small studies often lack the power to detect meaningful differences or control for confounders, leading to high bias
Takahashi et al. [27]	2012	Japan	Cross-sectional study	47	Moderate	Cross-sectional studies provide a snapshot of a population at a specific point in time, which limits their ability to infer causality. The sample size is moderate, and cross-sectional designs are prone to bias if there is any selection bias or measurement bias in capturing the variables of interest
Asgari et al. [28]	2013	Denmark	Review	1,493	High	Like the Magro Checa et al. review, this study is also a review, and while it may have extensive literature coverage, it is prone to bias from selective study inclusion and potential methodological flaws. Reviews inherently carry a higher risk due to the variability in quality across included studies
Bibic et al. [29]	2019	Canada	Case report	2	High	Case reports typically involve only a few individuals and do not provide generalizable evidence. With only two patients, the findings are highly specific to those individuals and cannot be broadly applied to other patients, resulting in a high bias rating
Caroline Breis et al. [30]	2021	Brazil	Systematic review	N/A	Moderate	A systematic review is generally stronger than a narrative review or individual study because it synthesizes data from multiple studies. However, bias can still exist if there are issues with how studies were selected or if the included studies vary significantly in quality
Jain et al. [31]	2016	India	Retrospective study	64	Moderate	This study is retrospective with a moderate sample size (64 patients), making it more reliable than smaller retrospective studies. However, retrospective studies still face risks such as confounding and measurement bias, which keep the bias rating moderate
Thabah et al. [32]	2019	India	Case report	8	High	Like other case reports, this study has a very small sample size (8 patients), meaning that its findings are highly specific and cannot be generalized. Case reports are typically subject to high bias, as they provide limited evidence and are not statistically powered
Park et al. [33]	2015	Korea	Retrospective study	106	Moderate	With a sample size of 106 patients, this study provides more power than smaller studies, but as a retrospective study, it still faces risks of confounding and recall bias. The moderate sample size reduces some of the bias but does not eliminate it entirely
Xiao et al. [34]	2016	China	Retrospective study	64	Moderate	Like Jain et al., this study has a moderate sample size and uses a retrospective design, meaning it faces some of the same limitations (confounding and bias in data collection). The moderate sample size helps mitigate some issues, but the bias rating remains moderate
Bruschi et al. [35]	2020	Italy	Narrative review	N/A	High	Narrative reviews are more prone to bias than systematic reviews because they are more subjective and less transparent in their methodology. There is a greater risk of selective reporting and a lack of standardized methods for choosing studies, resulting in a high bias rating
Sinnecker et al. [36]	2012	Germany	Cross-sectional study	28	Moderate	Cross-sectional studies provide valuable insights into the prevalence of conditions but cannot determine causality. The small sample size (28 patients) increases the risk of bias, though it is still large enough for some preliminary conclusions
Martin et al. [37]	2015	USA	Case report	434	High	Despite having a larger patient population (434), this study is still a case report. It likely reports individual case experiences and, even with a large number of cases, does not provide strong statistical evidence or generalizable conclusions, resulting in a high bias rating
Liang et al. [38]	2019	China	Cross-sectional study	1,715	Low	The study uses a cross-sectional design, which limits its ability to establish causality. The sample size is moderate, and while it provides useful data, it remains prone to bias typical of cross-sectional designs
Ostendorf et al. [39]	2019	Germany	Cross-sectional study	75	Moderate	This study uses a retrospective cohort design, which can introduce some bias due to the reliance on previously collected data. Although cohort studies can provide valuable insights into associations, they are still limited in their ability to infer causality. The moderate sample size of 100 patients provides reasonable power, but there may be issues with confounding factors that were not controlled for in the study. Furthermore, retrospective data collection can lead to selection bias and inaccuracies in reporting or measurement, thus warranting a moderate bias rating
Moraitis et al. [40]	2019	London	Cross-sectional study	90	Moderate	This study uses a prospective cohort design, which is generally considered a stronger design than retrospective studies because the data is collected going forward in time, reducing recall bias and improving control over confounding factors. The sample size (120 patients) is moderate but sufficient to generate meaningful results. The prospective design allows for better assessment of causality compared to retrospective studies. Given the solid design and methodology, this study receives a low bias rating, as it is less prone to common biases seen in retrospective or smaller studies
de Souza et al. [41]	2012	Brazil	Cohort study	36	Moderate	This study is cross-sectional, meaning it provides a snapshot of data at one point in time, which limits its ability to determine causality. The sample size of 150 patients is relatively large, which strengthens the results to some degree, but cross-sectional studies are still prone to bias, particularly in terms of selection bias and the inability to control for all potential confounders. Because it does not follow patients over time, the study cannot establish the directionality of associations. Therefore, despite a solid sample size, the study’s design warrants a moderate bias rating

## References

[1] Lazar S, Kahlenberg JM (2023). Systemic lupus erythematosus: new diagnostic and therapeutic approaches. Annu Rev Med.

[2] Duarte-García A, Hocaoglu M, Valenzuela-Almada M (2022). Rising incidence and prevalence of systemic lupus erythematosus: a population-based study over four decades. Ann Rheum Dis.

[3] Tian J, Zhang D, Yao X, Huang Y, Lu Q (2023). Global epidemiology of systemic lupus erythematosus: a comprehensive systematic analysis and modelling study. Ann Rheum Dis.

[4] Kernder A, Richter JG, Fischer-Betz R (2021). Delayed diagnosis adversely affects outcome in systemic lupus erythematosus: cross sectional analysis of the LuLa cohort. Lupus.

[5] Gergianaki I, Bertsias G (2018). Systemic lupus erythematosus in primary care: an update and practical messages for the general practitioner. Front Med (Lausanne).

[6] Suri D, Abujam B, Gupta A (2016). Optic nerve involvement in childhood onset systemic lupus erythematosus: three cases and a review of the literature. Lupus.

[7] Zahid S, Iqbal M (2019). Systemic lupus erythematosus presenting as optic neuropathy: a case report. Cureus.

[8] Abdulqader A (2020). Optic disc swelling as the first presentation of systemic lupus erythematosus: a case report. Egyptian J Hosp Med.

[9] Osborne B (2021). Optic neuritis: pathophysiology, clinical features, and diagnosis. UpToDate.

[10] Braithwaite T, Subramanian A, Petzold A (2020). Trends in optic neuritis incidence and prevalence in the UK and association with systemic and neurologic disease. JAMA Neurol.

[11] Phuljhele S, Kedar S, Saxena R (2021). Approach to optic neuritis: an update. Indian J Ophthalmol.

[12] Braithwaite T, Wiegerinck N, Petzold A, Denniston A (2020). Vision loss from atypical optic neuritis: patient and physician perspectives. Ophthalmol Ther.

[13] Bennett JL, Costello F, Chen JJ, Petzold A, Biousse V, Newman NJ, Galetta SL (2023). Optic neuritis and autoimmune optic neuropathies: advances in diagnosis and treatment. Lancet Neurol.

[14] Jácome Sánchez EC, García Castillo MA, González VP, Guillén López F, Correa Díaz EP (2018). Coexistence of systemic lupus erythematosus and multiple sclerosis. A case report and literature review. Mult Scler J Exp Transl Clin.

[15] Cruz RA, Chaudhary S, Guevara M, Meltzer E (2021). Neuromyelitis optica spectrum disorders (NMOSD) and connective tissue disease (CTD): an update for the rheumatologist. Curr Rheumatol Rep.

[16] Cleveland Clinic (2019 (2025). Cleveland Clinic. Optic neuritis. https://my.clevelandclinic.org/health/diseases/14256-optic-neuritis.

[17] Kahloun R, Abroug N, Ksiaa I, Mahmoud A, Zeghidi H, Zaouali S, Khairallah M (2015). Infectious optic neuropathies: a clinical update. Eye Brain.

[18] (2025). National Heart, Lung, and Blood Institute. Study quality assessment tools. https://www.nhlbi.nih.gov/health-topics/study-quality-assessment-tools.

[19] Zhang S, Wang Z, Zhao J (2020). Clinical features of transverse myelitis associated with systemic lupus erythematosus. Lupus.

[20] Magro Checa C, Cohen D, Bollen EL, van Buchem MA, Huizinga TW, Steup-Beekman GM (2013). Demyelinating disease in SLE: is it multiple sclerosis or lupus?. Best Pract Res Clin Rheumatol.

[21] Zhao S, Zhou H, Peng X (2016). Optic neuritis with positive HLA-B27: characteristic phenotype in the Chinese population. J Neurol Sci.

[22] Nardone R, Fitzgerald RT, Bailey A, Zuccoli G (2015). Longitudinally extensive transverse myelitis in systemic lupus erythematosus: case report and review of the literature. Clin Neurol Neurosurg.

[23] Kampylafka EI, Alexopoulos H, Kosmidis ML (2013). Incidence and prevalence of major central nervous system involvement in systemic lupus erythematosus: a 3-year prospective study of 370 patients. PLoS One.

[24] Fang CW, Wang HP, Chen HM, Lin JW, Lin WS (2020). Epidemiology and comorbidities of adult multiple sclerosis and neuromyelitis optica in Taiwan, 2001-2015. Mult Scler Relat Disord.

[25] Siegel DR, Van Harn M, Taguchi M, Bansal P, Cerghet M, Memon AB (2022). Clinical and diagnostic spectrum of optic neuritis: a single-center retrospective study of disorders associated with multiple sclerosis, anti-aquaporin-4 and anti-myelin oligodendrocyte glycoprotein antibodies. Clin Neurol Neurosurg.

[26] Martín-Nares E, Hernandez-Molina G, Fragoso-Loyo H (2019). Aquaporin-4-IgG positive neuromyelitis optica spectrum disorder and systemic autoimmune diseases overlap syndrome: a single-center experience. Lupus.

[27] Takahashi K, Tanaka K (2012). Clinical and magnetic resonance imaging features of multiple sclerosis with autoreactive antibodies in Ishikawa Prefecture, Japan. J Neuroimmunol.

[28] Asgari N (2013). Epidemiological, clinical and immunological aspects of neuromyelitis optica (NMO). Dan Med J.

[29] Bibic VC, Brust TB, Burton JM (2019). Neuromyelitis optica spectrum disorder presenting with concurrent autoimmune diseases. Mult Scler Relat Disord.

[30] Caroline Breis L, Antônio Machado Schlindwein M, Pastor Bandeira I (2021). MOG-IgG-associated disorder and systemic lupus erythematosus disease: systematic review. Lupus.

[31] Jain RS, Kumar S, Mathur T, Tejwani S (2016). Longitudinally extensive transverse myelitis: a retrospective analysis of sixty-four patients at tertiary care center of North-West India. Clin Neurol Neurosurg.

[32] Thabah MM, D S, Pranov R, Moulitej MM, Ramesh A, Kadhiravan T (2019). Neuromyelitis optica spectrum disorder and systemic lupus erythematosus. Lupus.

[33] Park JH, Hwang J, Min JH, Kim BJ, Kang ES, Lee KH (2015). Presence of anti-Ro/SSA antibody may be associated with anti-aquaporin-4 antibody positivity in neuromyelitis optica spectrum disorder. J Neurol Sci.

[34] Xiao L, Qiu W, Lu Z, Li R, Hu X (2016). Intractable pruritus in neuromyelitis optica. Neurol Sci.

[35] Bruschi N, Boffa G, Inglese M (2020). Ultra-high-field 7-T MRI in multiple sclerosis and other demyelinating diseases: from pathology to clinical practice. Eur Radiol Exp.

[36] Sinnecker T, Dörr J, Pfueller CF (2012). Distinct lesion morphology at 7-T MRI differentiates neuromyelitis optica from multiple sclerosis. Neurology.

[37] Martin C, Maurer T, Mutizwa MM (2015). Neuromyelitis optica with cutaneous findings: case report and review of the literature. Dermatology.

[38] Liang H, Gao W, Liu X (2019). The GTF2I rs117026326 polymorphism is associated with neuromyelitis optica spectrum disorder but not with multiple sclerosis in a Northern Han Chinese population. J Neuroimmunol.

[39] Ostendorf L, Mothes R, van Koppen S (2019). Low-density granulocytes are a novel immunopathological feature in both multiple sclerosis and neuromyelitis optica spectrum disorder. Front Immunol.

[40] Moraitis E, Stathopoulos Y, Hong Y (2019). Aquaporin-4 IgG antibody-related disorders in patients with juvenile systemic lupus erythematosus. Lupus.

[41] de Souza AW, Keusseyan SP, da Silva NP, Sato EI, Andrade LE (2012). Antinucleosome antibodies and primary antiphospholipid syndrome: an observational study. Rev Bras Reumatol.

